# Supplementary material for: Interfacial Coupling Controls Molecular Epitaxy of HMTP on Graphene/SiC
Source: ACS Appl Mater Interfaces. 2026 Apr 16;18(16):23839–47. doi: 10.1021/acsami.6c03070 (PMC13133772; doi:10.1021/acsami.6c03070)
Supplement: Supplementary file 1 [file am6c03070_si_001.pdf]

SUPPORTING INFORMATION

Interfacial Coupling Controls Molecular Epitaxy of HMTP on Graphene/SiC

Devanshu Varshney,[1&] *Pavel Procházka,*[2&] *Veronika Stará,*[2] *Mykhailo Shestopalov,*[3] *Jan Kunc,*[3*] *Jiří Novák,*[1*] *Jan Čechal*[2,4*]

[1]Department of Condensed Matter Physics, Faculty of Science, Masaryk University, Kotlářská 2, 61137 Brno, Czech Republic

[2]CEITEC - Central European Institute of Technology, Brno University of Technology, Purkyňova 123, 612 00 Brno, Czech Republic.

[3] Charles University, Faculty of Mathematics and Physics, Institute of Physics, Ke Karlovu 5, 121 16, Prague 2, Czech Republic

[4] Institute of Physical Engineering, Brno University of Technology, Technická 2896/2, 616 69 Brno, Czech Republic.

[&]These authors contributed equally.

*Corresponding authors' emails: jan.kunc@matfyz.cuni.cz (J.K.); novak@physics.muni.cz (J.N.); cechal@fme.vutbr.cz (J.Č.)

CONTENTS:

1. Raman Spectroscopy characterization of SLG and buffer layer substrates

2. XRD and AFM measured on samples with a coexisting buffer layer and SLG

3. Full-scale symmetric scan

4. The large-scale AFM images measured on the buffer and SLG

5. LEED model of graphene on SiC and moiré structure

6. LEED model of HMTP on graphene

7. LEEM dark-field analysis of HMTP on SLG

8. LEED single-domain diffraction of HMTP on the buffer layer

1. Raman Spectroscopy characterization of SLG and buffer layer substrates

As detailed below, a typical SLG shows a Lorentzian 2D peak with a FWHM of 32–40 cm^{-1} and an integrated 2D-to-G peak intensity ratio of 1.8 ± 0.3, typical fingerprints of SLG. The quasi-freestanding SLG shows a narrower 2D peak with FWHM of 23–33 cm^{-1}, and patches of bilayer graphene with FWHM in the 50–56 cm^{-1} range.

1A. Comparison of the SLG and quasi-freestanding SLG samples:

The ratio of G to D peak integrated intensities (Figure S1a) is proportional to the graphene grain size. It is ~2 for SLG because the underlying buffer layer contributes to the enhanced D peak intensity. The G to D peak ratio approaches ~5 for quasi-freestanding SLG, which corresponds to the grain size of about 100 nm when using 532 nm laser excitation.[1] The ratios of integrated 2D to G peak intensity of 1.8 ± 0.3 and 4.5 ± 0.5 given in Figure S1b are characteristic to SLG and quasi-freestanding SLG, respectively. The strain given in Figure S1c was determined from the G and 2D peak positions. The quasi-freestanding SLG shows a partially relaxed strain compared to SLG samples.

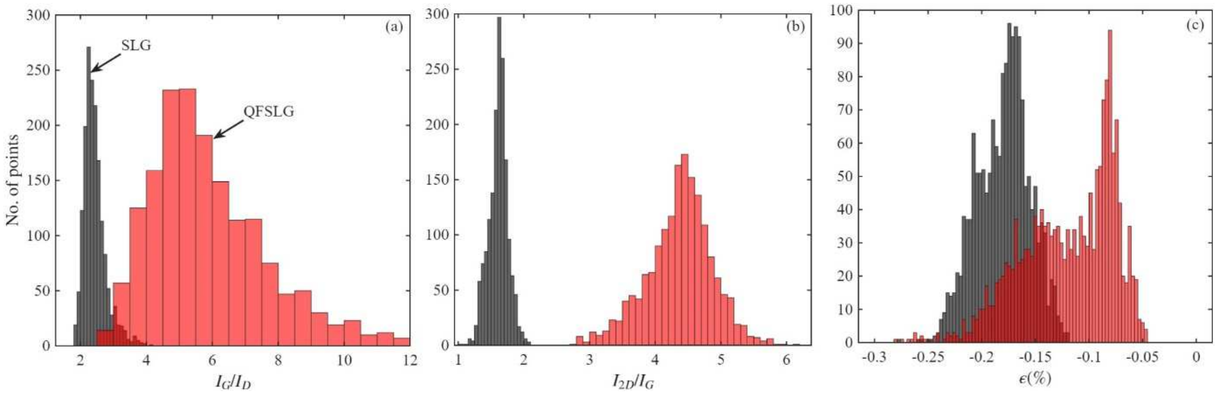

Figure S1: Comparison of Raman peak intensities measured on the SLG and quasi-freestanding SLG (QFSLG) samples: (a) G-to-D peak ratio and (b) 2D-to-G peak ratio. (c) Calculated elastic strain in the layer.

The 2D peak position is governed mostly by mechanical strain. The redshifted 2D peak in Figure S2a reflects a partially relaxed strain of the quasi-freestanding SLG. The improved quality of quasi-freestanding SLG compared to SLG is also revealed by the improved FWHM of 2D peak from 33 ± 2 cm^{-1} for SLG to 23 ± 2 cm^{-1} for quasi-freestanding SLG (Figure S2b). The FWHM of the 2D peak is a fingerprint of inhomogeneous strain on a sub-micrometer length scale.

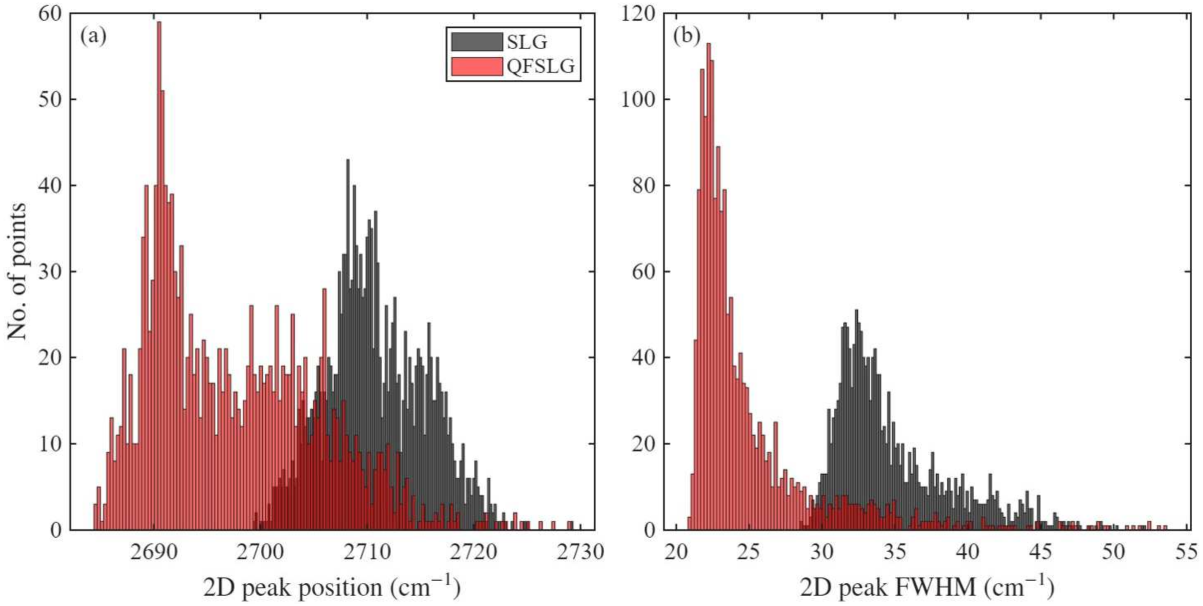

Figure S2: Comparison of Raman 2D peak position and FWHM measured on the SLG and quasi-freestanding SLG (QFSLG) samples: (a) 2D peak position and (b) 2D peak FWHM.

1B. Comparison of buffer layer samples

The buffer layer before the hydrogen intercalation is the precursor used to fabricate the quasi-freestanding SLG sample. A small ratio of the G to D peak integrated intensities 1.2 ± 0.2 (Figure S3a) reveals a small grain size of the buffer layer around 25 ± 5 nm. The broad G peak in Figure S3b indicates a largely distorted graphene-like lattice. The nearly zero 2D peak intensity relative to the G peak intensity (Figure S3c) indicates an undeveloped graphene electronic band structure.

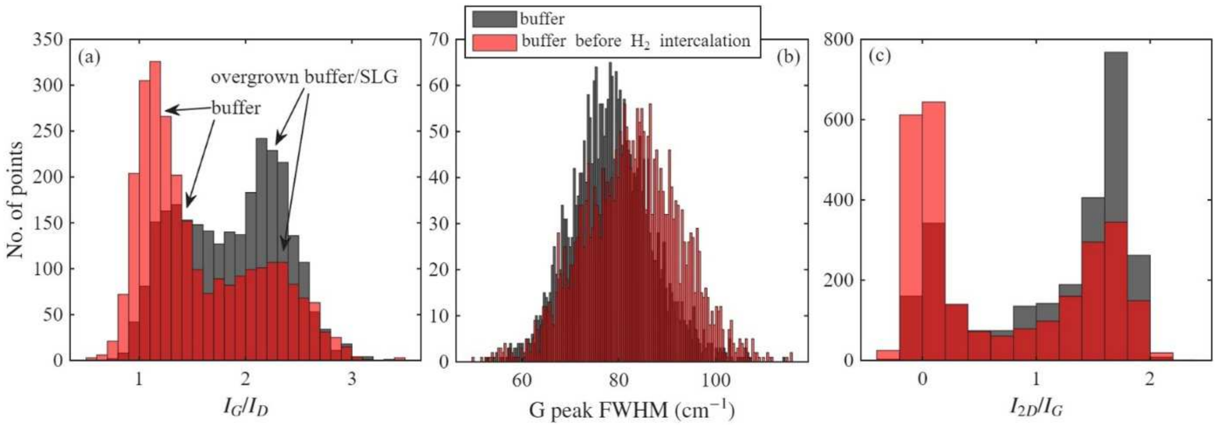

Figure S3: Comparison of Raman peak signals measured on the two distinct samples with the buffer layer: one was used for HMTP deposition, and the second for preparation of quasi-freestanding SLG; this shows a sample-to-sample variability of buffer layers. (a) G-to-D peak ratio, (b) FWHM of the G peak, and (c) 2D-to-G peak ratio.

1C Sample homogeneity and typical Raman spectra

To assess the homogeneity of the SLG samples, Raman maps were clustered into five areas and further reduced to typical graphene allotropes, as shown in Figures S4a, d, and g. The corresponding 2D peak spectra are shown in Figures S4b, e, and h, and the number of 2D peak components can be determined from the number of minima in the second derivative given in Figures S4c, f, and i.

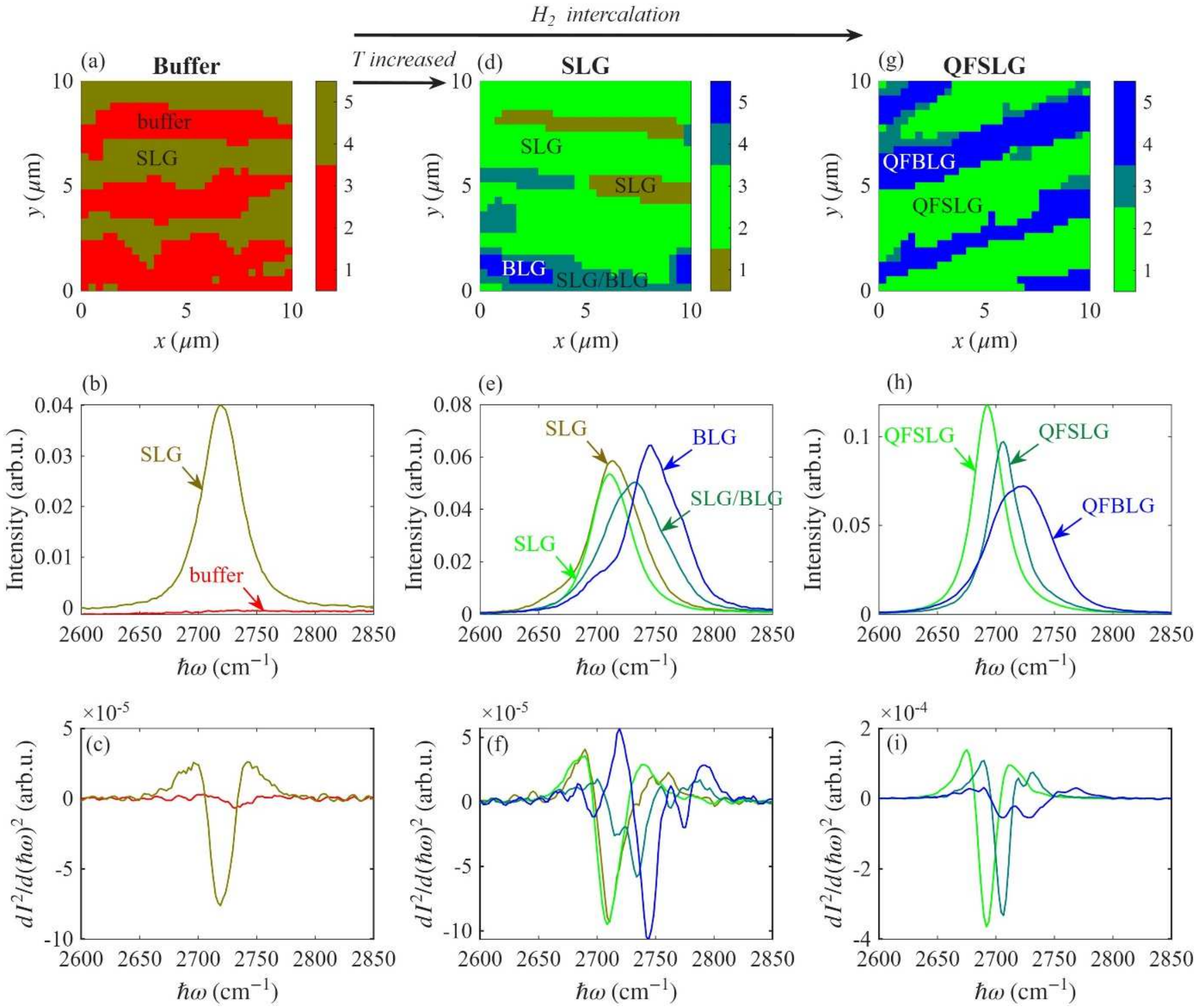

Figure S4: (a), (d), and (g) Raman maps clustered into five different areas on distinct substrates: (a) the buffer layer, (d) SLG, and (g) quasi-freestanding SLG (QFSLG). The clusters are color-coded as follows red: the buffer layer; dark yellow: strained SLG or mixture of SLG and bilayer graphene; light green: SLG in (d) and QFSLG in (g); dark cyan: transition between SLG and bilayer graphene (BLG) or QFSLG to BFLG in (d) and (g), respectively; blue: BLG and QFBLG in (d) and (g), respectively. (b), (e), and (h) Raman spectra of the 2D peak region associated with clusters in (a), (d), and (g) measured on the (b) buffer, (e) SLG, and (h) quasi-freestanding SLG (QFSLG). The spectra are averaged within each of the five clusters. (c), (f), and (i) The second derivative of the spectra shows the number of 2D peak components. A single-component 2D peak represents SLG; the four-component 2D peak represents bilayer graphene. Bilayer graphene typically grows at SiC step edges.

2. XRD and AFM measured on samples with a coexisting buffer layer and SLG

The diffraction data for the substrate with a coexisting buffer layer and SLG (approximate 2:1 coverage ratio), shown in Figure S5, correspond to the sum of the diffraction patterns from samples with only the buffer layer and only SLG, respectively. The pole figure of HMTP $\{10\bar{1}1\}$ shows two sets of six sharp spots at radial and azimuthal positions identical to the SLG sample (Figure 2 in the main text), together with a slightly enhanced background. The orientational order characteristic of the HMTP on SLG dominates the scattering pattern, despite the buffer layer covering most of the substrate. The detailed analysis of the in-plane directional ordering was done by azimuthal scans probing HMTP $\{10\bar{1}1\}$ planes radially tilted out of surface plane by 31.7° (Figures S5b and c). Also, here, we observe sharp and intense HMTP $\{10\bar{1}1\}$ reflections offset by ±19.1° out of nearest SiC $\{11\bar{2}0\}$ reflections, corresponding to the intense spots in the pole figure, and indicating the same rotation angle of the HMTP lattice as observed for the SLG sample. Additionally, there are less intense and broader peaks corresponding to HMTP on the buffer layer, where HMTP $\{10\bar{1}1\}$ reflections are offset by ±11° out of nearest SiC $\{11\bar{2}0\}$ reflections. Their intensity is suppressed, and peaks are azimuthally shifted relative to the corresponding buffer-layer peaks from ±8.2° to ±11.0° (see Figure 2 in the main text). This suggests that the preferential in-plane rotation of the HMTP domains on the buffer is driven by the amount of contact with SLG domains with which they coexist.

The AFM images of the HMTP film grown on the sample with coexisting buffer and SLG are shown in Figure S6. A relatively flat percolated HMTP film decorates the SLG part that dendritically grows from SiC surface step edges, while small islands cover the buffer, with diameters in the hundreds of nm. This picture is consistent with AFM images of the SLG and buffer samples (Figure 4 in the main text and Figure S8), thus highlighting the coexistence of

the two types of HMTP film morphologies next to each other on the SiC substrate with coexisting buffer and SLG areas.

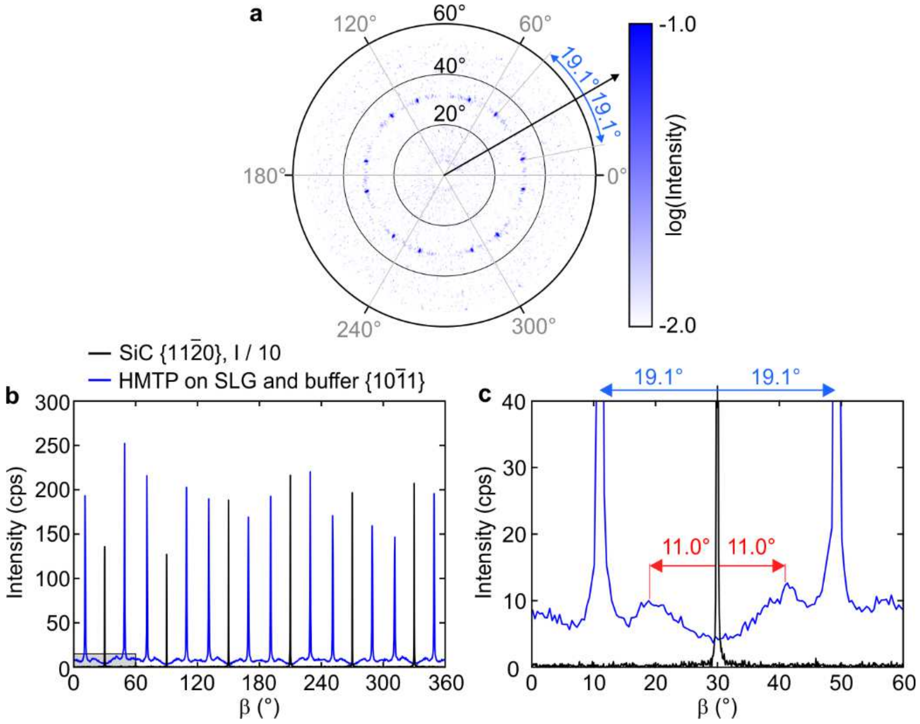

Figure S5: (a) Pole figure of HMTP $\{10\bar{1}1\}$ reflections and (b) azimuthal (β) scan of the HMTP $\{10\bar{1}1\}$ reflections at the polar angle 31.7° for an HMTP film on a substrate with a coexisting buffer layer and SLG (approximate 2:1 coverage ratio). In the azimuthal scans, the data for HMTP $\{10\bar{1}1\}$ (blue) are shown together with SiC substrate $\{11\bar{2}0\}$ in-plane reflections (black). The azimuthal angle β was aligned using the SiC $\{11\bar{2}0\}$ reflections as reference. The SiC intensity is downscaled by a factor of 10 for clarity. (c) An enlarged view of the azimuthal scans.

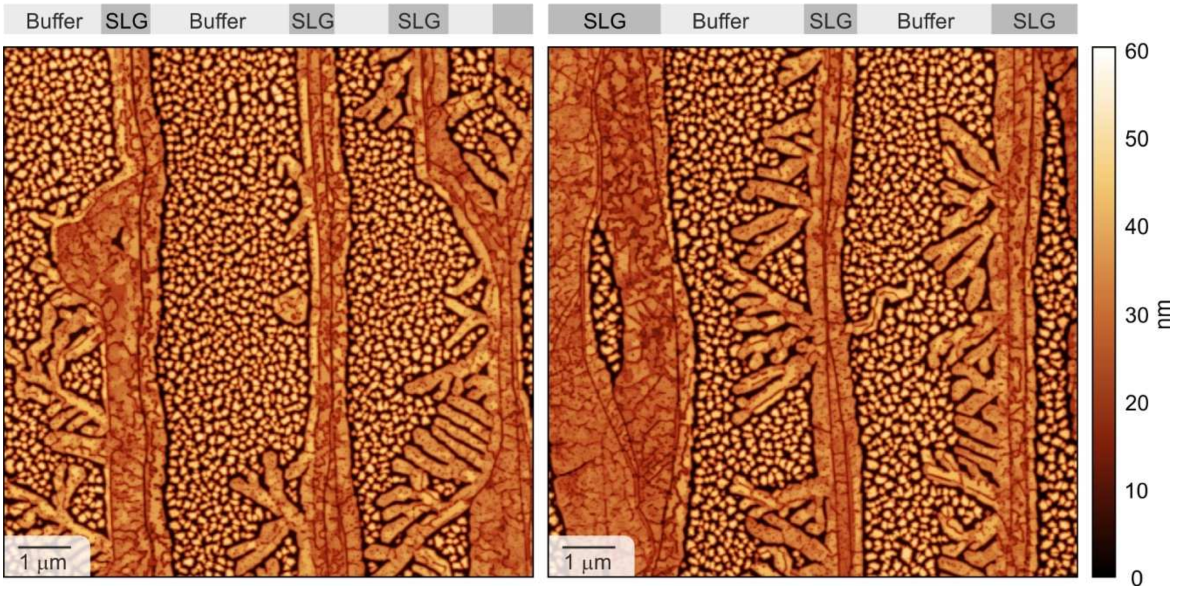

Figure S6: Large-scale AFM images of HMTP on a sample with coexisting SLG and the buffer layer (estimated coverage ratio 1:2). Identification of the respective areas is given on top of the figure.

3. Full-scale symmetric scan

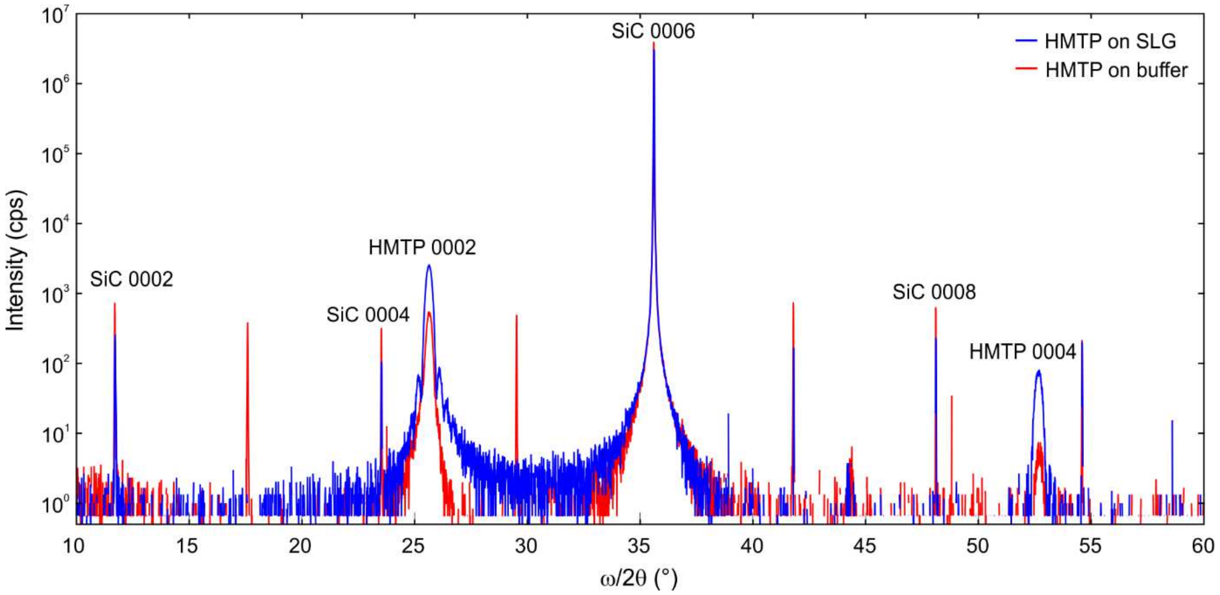

Figure S7: Overview of a symmetric ω/2θ scan for HMTP thin film on SLG (red) and the buffer layer (black). The marked peaks correspond to HMTP $000l$ and SiC $000l$ reflections from Cu Kα_1 radiation. The unmarked intense peaks originate from SiC $000l$ diffraction of higher harmonics.

4. The large-scale AFM images measured on the buffer and SLG

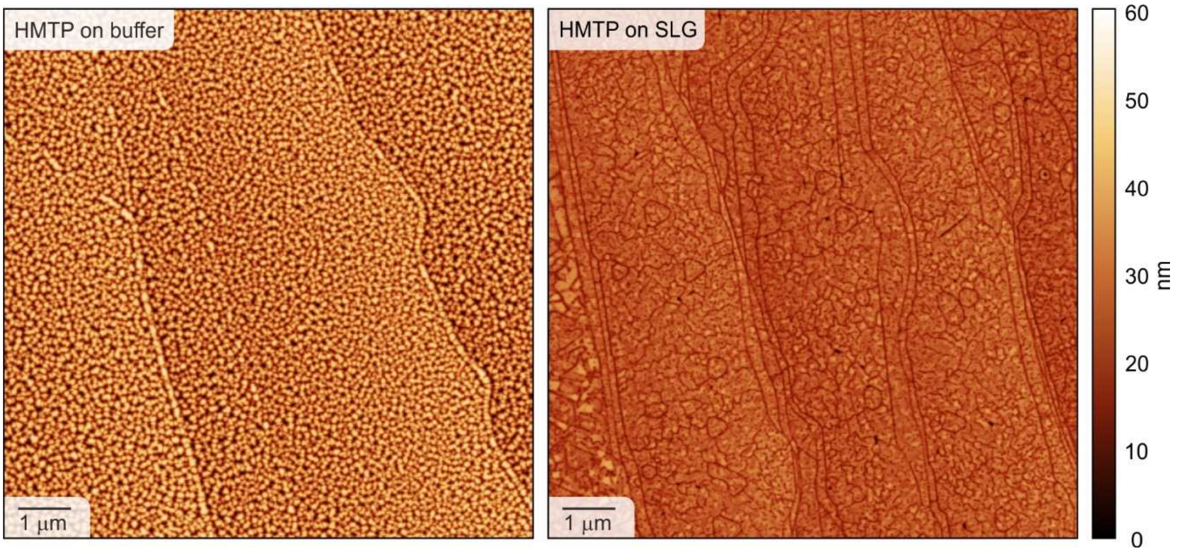

Figure S8: Large-scale AFM images of the HMTP film on the buffer layer and SLG associated with Figure 4 in the main text.

5. LEED model of graphene on SiC and moiré structure

Figure S9a illustrates the diffraction model of epitaxial graphene on SiC used to interpret the LEED features discussed in the main text. The diffraction pattern (Figure S9b) measured at 60 eV consists of graphene spots, SiC substrate spots, and additional satellite (moiré) spots arising from their relative lattice mismatch and rotational alignment. The corresponding model, generated in ProLEED Studio,[2] overlays the reciprocal lattices of graphene and SiC and reproduces the positions of the observed moiré spots.

To visualize the structural origin of these features, the real-space SiC and graphene lattices are shown together (Figure S9a) with their respective unit cells. The large-scale moiré superlattice emerges from the mismatch between the 2.46 Å graphene lattice and the 3.08 Å SiC lattice and corresponds to a $(6\sqrt{3} \times 6\sqrt{3})R30°$ reconstruction with a periodicity of approximately 32 Å, as shown in Figure S9c,d. An enlarged view of the moiré diffraction in Figure S9c shows the moiré unit cell and the reciprocal-lattice vectors responsible for the satellite spots observed in LEED. In the main text, only the large, higher-order moiré spots are visible, while the primary moiré spots remain too weak to be detected.

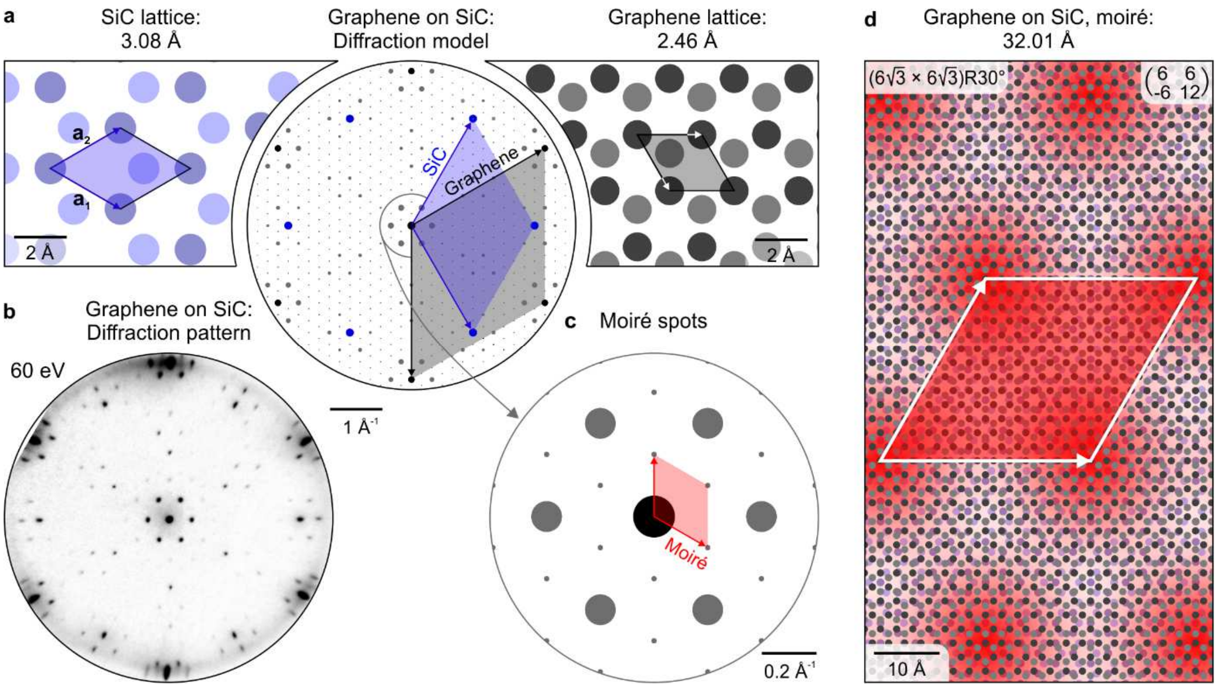

Figure S9: (a) Real-space SiC (3.08 Å) and graphene (2.46 Å) lattices with their unit cells. The ProLEED Studio diffraction model of graphene on SiC (center) shows the corresponding reciprocal lattice points of graphene (black) and SiC (blue). (b) Experimental LEED pattern measured at 60 eV. (c) Enlarged view of the diffraction model highlighting the moiré spots, with the moiré unit cell marked. (d) Real-space representation of the (6√3 × 6√3)R30° moiré superlattice with a periodicity of ~32 Å. The moiré spots originate from the relative lattice mismatch and 30° rotation between graphene and the SiC substrate.

6. LEED model of HMTP on graphene

Figure S10 summarizes the diffraction model used to interpret the HMTP overlayer on SLG. The full diffraction pattern in Figure S10a shows the combined contributions of the two symmetry-equivalent HMTP domains. A comparison with the simulated pattern confirms the assignment of the main diffraction spots. Single-domain diffraction patterns extracted from individual regions of the sample are shown in Figure S10b. Their corresponding simulated patterns in Figure S10c reproduce the positions of the domain-specific spots and verify that both domains adopt the same commensurate superstructure with different in-plane orientations.

Figure S10d presents the real-space structural model of the HMTP overlayer with respect to the graphene lattice for each domain. The superstructure corresponds to the $\begin{pmatrix} 4 & 2 \\ 6 & -4 \end{pmatrix}$ matrix notation and describes a $2\sqrt{7} \times 2\sqrt{7}$ R19.1° commensurate unit cell. The two equivalent HMTP orientations differ only by their rotational alignment relative to graphene.

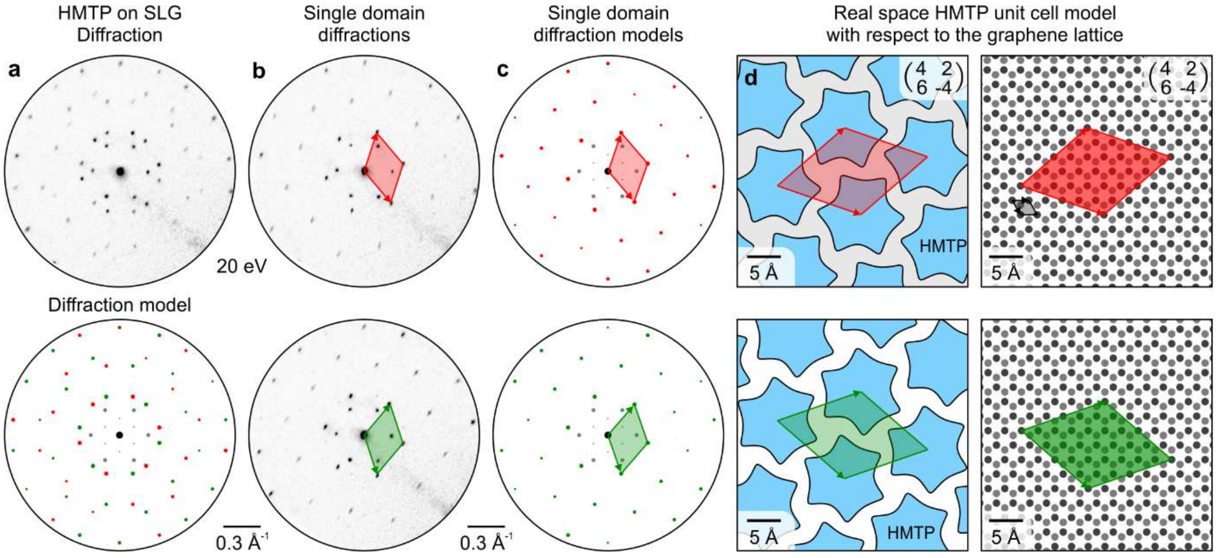

Figure S10: HMTP diffraction model on SLG. (a) Experimental HMTP diffraction pattern on SLG at 20 eV together with its simulated model. (b) Single-domain diffraction patterns extracted from two HMTP domains. (c) Corresponding simulated patterns for the two domain

orientations. (d) Real-space structural model of the HMTP unit cell relative to the graphene lattice for both orientations, described by the $\begin{pmatrix} 4 & 2 \\ 6 & -4 \end{pmatrix}$ matrix notation corresponding to a $2\sqrt{7} \times 2\sqrt{7}$ R19.1° superstructure.

7. LEEM dark-field analysis of HMTP on SLG

Figure S11 shows the individual dark-field images used to construct the composite DF presented in Figure 5f of the main text. Each dark-field image was obtained by selecting one of the two HMTP diffraction spots highlighted in the LEED pattern, isolating a single HMTP domain orientation on SLG. The resulting images, together with their color-coded composite, confirm that ordered HMTP domains form exclusively on SLG, while no crystalline contrast is detected on the buffer layer.

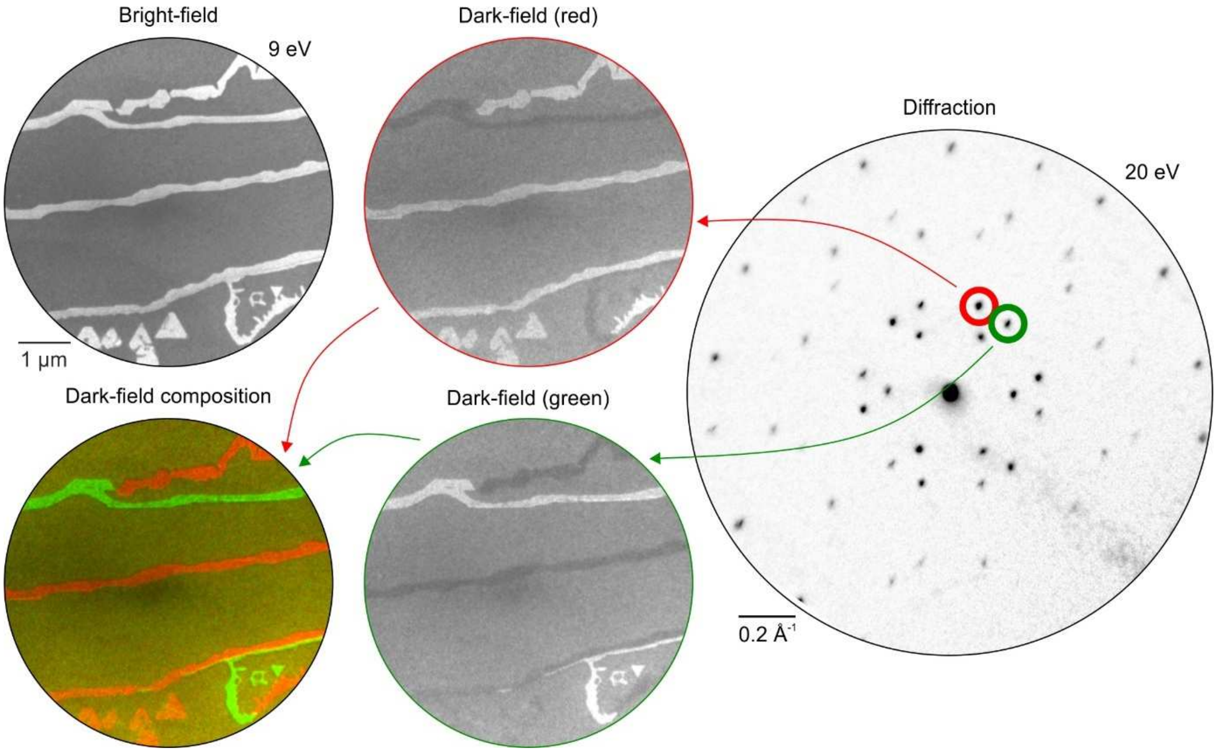

Figure S11: Individual dark-field images underlying the composite shown in Figure 5f of the main text. The figure includes the bright-field image (9 eV), the LEED pattern (20 eV) with the selected diffraction spots, and the corresponding dark-field images for the two HMTP orientations on SLG. The color-coded composite maps the spatial distribution of both orientations. Ordered HMTP is observed only on SLG, with no crystalline contrast on the buffer layer.

8. LEED single-domain diffraction of HMTP on the buffer layer

As shown in Figure S12, single-domain LEED patterns measured on the buffer layer display only the characteristic moiré spots of graphene/SiC. No additional diffraction spots associated with HMTP are observed, in contrast to the ordered domains on SLG. This confirms that HMTP does not form a crystalline overlayer on the buffer layer.

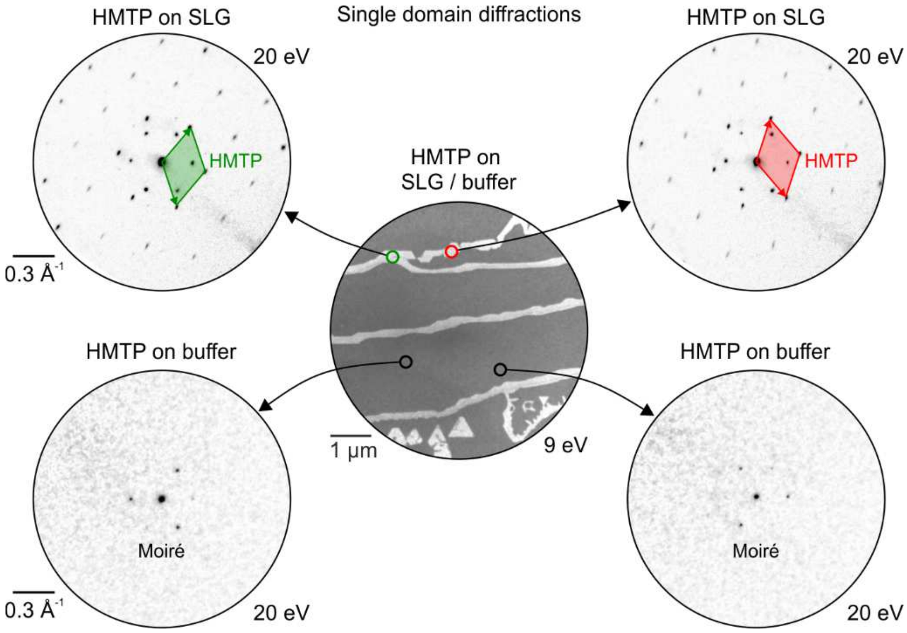

Figure S12: Single-domain diffraction measurements of HMTP on SLG and the buffer layer. The bright-field image (center) indicates the probed positions. On SLG (top), two distinct HMTP domain orientations produce sharp diffraction spots. On the buffer layer (bottom), only the substrate-related moiré spots are present, with no additional diffraction attributable to HMTP.

REFERENCES

(1) Kunc, J.; Rejhon, M. Raman 2D Peak Line Shape in Epigraphene on SiC. *Appl. Sci.* **2020**, *10* (7), 2354. https://doi.org/10.3390/app10072354.

(2) Procházka, P.; Čechal, J. ProLEED Studio : Software for Modeling Low-Energy Electron Diffraction Patterns. *J. Appl. Crystallogr.* **2024**, *57*, 187. https://doi.org/10.1107/S1600576723010312.
